# ﻿*Marsupellabrasiliensis* sp. nov. (Gymnomitriaceae, Marchantiophyta) from Brazil – the distribution of sect. *Stolonicaulon* in Neotropics is now confirmed

**DOI:** 10.3897/phytokeys.226.103975

**Published:** 2023-05-12

**Authors:** Vadim A. Bakalin, Yulia D. Maltseva, Alfons Schäfer-Verwimp, Seung Se Choi

**Affiliations:** 1 Laboratory of Cryptogamic Biota, Botanical Garden-Institute FEB RAS, Makovskogo Street 142, 690024 Vladivostok, Russia Laboratory of Cryptogamic Biota, Botanical Garden-Institute FEB RAS Vladivostok Russia; 2 Mittlere Letten 11, 88634 Herdwangen-Schönach, Germany Unaffiliated Herdwangen-Schönach Germany; 3 Team of National Ecosystem Survey, National Institute of Ecology, Seocheon 33657, Republic of Korea Team of National Ecosystem Survey, National Institute of Ecology Seocheon Republic of Korea

**Keywords:** Distribution patterns, endemics, Latin America, Marchantiophyta, *Marsupella*, molecular-genetic

## Abstract

The specimen previously identified as *Marsupellamicrophylla* from Brazil is reassessed and described as a new species, *M.brasiliensis*. The new species is characterized by paroicous inflorescence, bispiral elaters, scale-like, commonly unlobed leaves and very small leaf cells. Descriptions and drawings are provided along with a corresponding discussion of the morphological peculiarity of the new species. *Marsupellabrasiliensis* belongs to sect. Stolonicaulon, and the distribution of Marsupellasect.Stolonicaulon in the New World is confirmed. The infrageneric position of *M.microphylla* remains unresolved, and whether it belongs to the same section is still unclear.

## ﻿Introduction

Marsupellasect.Stolonicaulon (N. Kitag.) Váňa is a mysterious section that includes the smallest taxa of the genus and is characterized by a disjunct range, mainly in the tropical and subtropical mountains of Asia to Melanesia, as well as in the Venezuelan Andes and the mountain range of SE Brazil of Latin America (Bakalin et al. 2022). This section possesses a number of distinctive morphological features that are not characteristic of other members of the genus, such as regular underleaves and scale-like unlobed leaves. A recent revision of this section (Bakalin et al. 2022) showed that it contains sterile morphotypes similar to those in other families; for example, the regular underleaves and remote leaves resemble *Cephaloziella* (*Marsupellapraetermissa* Bakalin & Vilnet, *M.taiwanica* Mamontov, Vilnet & Schäf.-Verw.) or deeply dissected conduplicate leaves, resemble small *Schizophyllopsis* Váňa & L. Söderstr. (*Marsupellaanastrophylloides* Bakalin, Vilnet & Maltseva). The cited revision (Bakalin et al. 2022) was limited by the integrative study of Asian taxa of the section. Therefore, the question arose whether it is correct to refer to sect. *StolonicaulonMarsupellamicrophylla* R.M. Schust., as suggested by Bakalin et al. (2019). Moreover, the latter taxon was originally placed in its own monotypic subgenus: Marsupellasubg.Nanocaulon R.M. Schust. (Schuster 1996) and then transferred to sect. Boeckiorum Müll. Frib. ex R.M. Schust. (Váňa 1999).

One of the authors (A.S.-V.) collected a specimen identified by J. Váňa as *Marsupellamicrophylla* in the Brazilian state of Rio de Janeiro. This specimen is now being investigated in our integrative research. The purpose of this account is to resolve the taxonomic position of this specimen and a review of the genetic relationships within Marsupellasect.Stolonicaulon.

## ﻿Materials and methods

### ﻿Taxon sampling

The specimen identified by J. Váňa as *Marsupellamicrophylla* (referred to in the report as specimen ASV15033) was studied by traditional morphological techniques, and plant morphology was compared with other taxa of sect. Stolonicaulon (N. Kitag.) Váňa, originally described and well known in Asia, and further treatments of *Marsupellamicrophylla* by Schuster (1978, 1996).

To compile the dataset for molecular phylogenetic analysis, we sequenced two loci (ITS 1‒2 and *trn*L‒F) from the specimen ASV15033 and added these sequences to the dataset from Bakalin et al. (2022). The outgroup for tree rooting remained unchanged (*Eremonotusmyriocarpus* (Carrington) Lindb. & Kaal.). Specimen voucher details, including GenBank accession numbers, are listed in Table 1.

**Table 1.** The list of voucher details and GenBank accession numbers for the specimens used in the phylogenetic analysis in the present paper. The newly obtained sequences are marked in bold.

Taxon	Specimen voucher	GenBank accession number	
		ITS 1‒2 nrDNA	trnL‒F cpDNA
*Eremonotusmyriocarpus* (Carrington) Lindb. & Kaal. ex Pearson	Russia, Karachaevo-Cherkessian Rep., N. Konstantinova, K446-6-05, 109, 615 (KPABG)	EU791839	EU791716
*Gymnomitrionbrevissimum* (Dumort.) Warnst.	Russia, Murmansk Prov., N. Konstantinova, G 8171 (KPABG)	EU791833	EU791711
*Gymnomitrioncorallioides* Taylor ex Carrington	Norway, Svalbard, N. Konstantinova, K155-04, 110, 103 (KPABG)	EU791826	EU791705
*Marsupellaaleutica* Mamontov, Vilnet, Konstant. & Bakalin	USA, Alaska, Schoﬁeld, 103, 958 (MO)	MH826408	MH822632
*Marsupellaanastrophylloides* Bakalin, Vilnet & Maltseva	Vietnam, Hà Giang Prov., V.A. Bakalin & K.G. Klimova, V-15-6-20 (VBGI)	OM480746	OM489480
*Marsupellaapertifolia* Steph.	Russia, Sakhalin Prov., V.A. Bakalin, K-79-2-15 (VBGI), 123, 501 (KPABG)	MH539834	MH539891
*Marsupellaapiculata* Schiffn.	Norway, Svalbard, N. Konstantinova, K93-1-06, 111, 840 (KPABG)	EU791819	EU791699
*Marsupellaaquatica* (Lindenb.) Schiffn.	Russia, Murmansk Prov., N. Konstantinova, 152/5-87, 6090 (KPABG)	EU791813	AF519201
*Marsupellaarctica* (Berggr.) Bryhn & Kaal.	Norway, Svalbard, N. Konstantinova, 128-04 (KPABG)	EU791815	EU791695
*Marsupellaboeckii* (Austin) Lindb. ex Kaal.	Russia, Murmansk Prov., N. Konstantinova, 367-2-00, 8184 (KPABG)	EU791816	EU791696
*Marsupellabolanderi* (Austin) Underw. **2**	USA, California Monterey CO, (KPABG)	MF521464	MF521476
*Marsupellabolanderi* (Austin) Underw. **1**	USA, Santa Yen Mts. St. Barbara, 38802 (KPABG)	MF521463	MF521475
*Marsupellabrasiliensis* Bakalin, Maltseva & Schäf.-Verw. sp. nov.	Brazil, Rio de Janeiro, Serra de Itatiaia, Schäfer-Verwimp & Verwimp, 15033, 15.10.1991	**OQ398709**	**OQ408447**
*Marsupellacondensata* (Ångstr. ex C.Hartm.) Lindb. ex Kaal.	Russia, Kamchatka Terr., V.A. Bakalin, K-60-30-15 (VBGI)	MH539844	MH539901
*Marsupelladisticha* Steph.	Japan, Deguchi, Yamaguchi, Bryophytes of Asia 170 (2000) (KPABG)	EU791824	EU791703
*Marsupellaemarginata* (Ehrh.) Dumort.	Russia, Buryatia Rep., N. Konstantinova, 23-4-02, 104, 411 (KPABG)	EU791811	EU791692
*Marsupellafunckii* (F. Weber & D. Mohr) Dumort.	Russia, Karachaevo-Cherkessian Rep., N. Konstantinova, K516-1-05, 109, 804 (KPABG)	EU791820	EU791700
*Marsupellakoreana* Bakalin & Fedosov	Republic of Korea, KyongNam Province, V.A. Bakalin, Kor-23-18-15 (VBGI)	MH539850	MH539907
*Marsupellapatens* (N.Kitag.) Bakalin & Fedosov	Japan, Fukuoka Pref., V.A. Bakalin, J-7-26a-14 (VBGI)	MH539846	MH539903
*Marsupellapseudofunckii* S.Hatt.	Japan, Yamanashi Pref., V.A. Bakalin, J-7-10-14 (VBGI)	MH539852	MH539909
*Marsupellasphacelata* (Giesecke ex Lindenb.) Dumort.	Russia, Kemerovo Prov., N. Konstantinova, 65/1-00 (KPABG)	EU791821	AF519200
*Marsupellasprucei* (Limpr.) Bernet	Russia, Kemerovo Prov., N. Konstantinova, 54-1-00, 101, 850 (KPABG)	EU791823	HQ833031
*Marsupellastoloniformis* N.Kitag.	Vietnam, Lao Cai Prov., V.A. Bakalin & K.G. Klimova, V-11-11-17 (VBGI)	MH539859	MH539916
*Marsupellasubemarginata* Bakalin & Fedosov	Japan, Yamanashi Pref., V.A. Bakalin, J-89-31-15 (VBGI), 123, 468 (KPABG)	MH539836	MH539893
*Marsupellataiwanica* Mamontov, Vilnet & Schäf.-Verw. **2**	China, Taiwan, Nantou Co., A. Schäfer-Verwimp 37663 (MHA, TAIE, JE, VBGI), 123, 642 (KPABG)	OM509627	OM515126
*Marsupellataiwanica* Mamontov, Vilnet & Schäf.-Verw. **1**	China, Taiwan, Chiayi Co., A. Schäfer-Verwimp 39136 (MHA, TAIE, JE, VBGI), 123, 545 (KPABG)	OM509628	OM515127
*Marsupellatubulosa* Steph.	Russia, Kamchatka Terr., V.A. Bakalin, K-66-7-15 (VBGI)	MH539860	MH539917
*Marsupellavermiformis* (R.M.Schust.) Bakalin & Fedosov **2**	Republic of Korea, Jeju Prov., S.S. Choi, 120911-1 (VBGI)	MH539858	MH539915
*Marsupellavermiformis* (R.M. Schust.) Bakalin & Fedosov **1**	Republic of Korea, Jeju Prov., S.S. Choi, 120911-2 (VBGI)	MH539857	MH539914
*Marsupellavietnamica* Bakalin & Fedosov	Vietnam, Lao Cai Prov., V.A. Bakalin, V-2-101-16 (VBGI)	MH539862	MH539919
*Marsupellayakushimensis* (Horik.) S.Hatt.	Republic of Korea, Gangwon Prov., S.S. Choi, 8347 (VBGI)	MH539864	MH539921
*Nardiacompressa* (Hook.) Gray	Canada, British Columbia, N. Konstantinova, A 97/1-95 (KPABG)	EU791837	AF519188
*Prasanthussuecicus* (Gottsche) Lindb.	Norway, Svalbard, N. Konstantinova, K121-5-06, 111, 821 (KPABG)	EU791825	EU791704

### ﻿DNA isolation, amplification, and sequencing

DNA was extracted from dried liverwort tissue using the NucleoSpin Plant II Kit (Macherey-Nagel, Germany). Ampliﬁcation was performed using an Encyclo Plus PCR kit (Evrogen, Moscow, Russia) with the primers listed in Table 2.

**Table 2.** Primers used in polymerase chain reaction (PCR) and cycle sequencing.

Locus	Sequence (5'-3')	Direction	Annealing temperature (°C)	Reference
ITS 1–2 nrDNA	CGGTTCGCCGCCGGTGACG	forward	68	Groth et al. 2002
ITS 1–2 nrDNA	CGTTGTGAGAAGTTCATTAAACC	forward	64	Feldberg et al. 2016
ITS 1–2 nrDNA	TCGTAACAAGGTTTCCGTAGGTG	forward	68	Hsiao et al. 1994
ITS 1–2 nrDNA	GATATGCTTAAACTCAGCGG	reverse	58	Milyutina et al. 2010
*trn*L–F cpDNA	CGAAATTGGTAGACGCTGCG	forward	62	Bakalin et al. 2021
*trn*L–F cpDNA	TGCCAGAAACCAGATTTGAAC	reverse	60	Bakalin et al. 2021

The polymerase chain reaction was performed in a total volume of 20 µl, including 1 µl of template DNA, 0.4 µl of Encyclo polymerase, 4 µl of Encyclo buffer, 0.4 µl of dNTP-mixture (included in Encyclo Plus PCR Kit), 13.4 µl (for *trn*L–F)/12.4 µl (for ITS 1–2) of double-distilled water (Evrogen, Moscow, Russia), 1 µl of dimethylsulfoxide/DMSO (for ITS 1–2) and 0.4 µl of each primer (forward and reverse, at a concentration of 5 pmol/µl). Polymerase chain reactions were carried out using the following program: 180 sec initial denaturation at 94 °C, followed by 35 cycles of 30 sec denaturation at 95 °C, 20 (for *trn*L–F) – 30, 35 sec (for ITS 1–2) annealing at 58 °C (*trn*L–F and ITS 1–2) and 60 °C (ITS 1–2) and 30 sec elongation at 72 °C. Final elongation was carried out in one 3-min step at 72 °C. Amplified fragments were visualized on 1% agarose TAE gels by EthBr staining and purified using the Cleanup Mini Kit (Evrogen, Moscow, Russia). The DNA was sequenced using the ABI PRISM BigDye Terminator Cycle Sequencing Ready Reaction Kit (Applied Biosystems, Waltham, MA, USA) with further analysis of the reaction products following the standard protocol on an automatic sequencer 3730 DNA Analyzer. (Applied Biosystems, Waltham, MA, USA) in the Genome Center (Engelhardt Institute of Molecular Biology, Russian Academy of Sciences, Moscow).

### ﻿Phylogenetic analyses

The alignments were compiled for the ITS 1–2 and *trn*L–F loci and aligned using MAFFT (Katoh and Standley 2013) with standard settings and then edited manually in BioEdit ver. 7.2.5 (Hall 1999). All positions of the final alignment were included in the phylogenetic analyses. Absent data at the ends of regions and missing loci were coded as missing data. Both datasets revealed congruence after preliminary phylogenetic analysis, so we combined them into a single ITS 1–2+*trn*L–F alignment for further analysis.

Phylogenetic trees were reconstructed using two approaches: maximum likelihood (ML) with IQ-tree ver. 1.6.12 (Nguyen et al. 2015) and Bayesian inference (BA) with MrBayes ver. 3.2.7 (Ronquist et al. 2012).

For the ML analysis, the best fitting evolutionary model of nucleotide substitutions according to the BIC value was TIM2e+R2 determined by ModelFinder (model-selection method which is implemented in IQ-tree) (Kalyaanamoorthy et al. 2017). Consensus trees were constructed with 1000 bootstrap replicates.

Bayesian analyses were performed by running two parallel analyses using the GTR+I+G model. The analysis consisted of four Markov chains. Chains were run for five million generations, and trees were sampled every 500^th^ generation. The first 2,500 trees in each run were discarded as burn-in; thereafter, 15,000 trees were sampled from both runs to produce a resulting tree. Bayesian posterior probabilities were calculated from the trees sampled after burn-in. The average standard deviation of split frequencies between two runs reached 0.0021 before the analysis was stopped.

To visualize molecular relationships within the Marsupellasect.Stolonicaulon we used TCS network inference method (Clement et al. 2002) in the PopART package (http://popart.otago.ac.nz/), accessed on 08 November 2019 (Leigh and Bryant 2015). The PopART program automatically removes positions having at least one N or a gap value from the consideration.

## ﻿Results

Newly obtained ITS 1‒2 and *trn*L‒F sequences from specimen ASV15033 were deposited in GenBank. The combined ITS 1‒2+*trn*L‒F alignment of the 33 specimens consisted of 1409 character sites: conservative sites ‒ 944 (67%); variable sites ‒ 420 (29.81%); and parsimony-informative sites ‒ 205 (14.55%).

The ML criterion recovered a bootstrap consensus tree with a log-likelihood = -6121.943. The arithmetic means of the log likelihoods in Bayesian analysis for each sampling run were -6145.17 and -6144.03.

**Figure 1.**
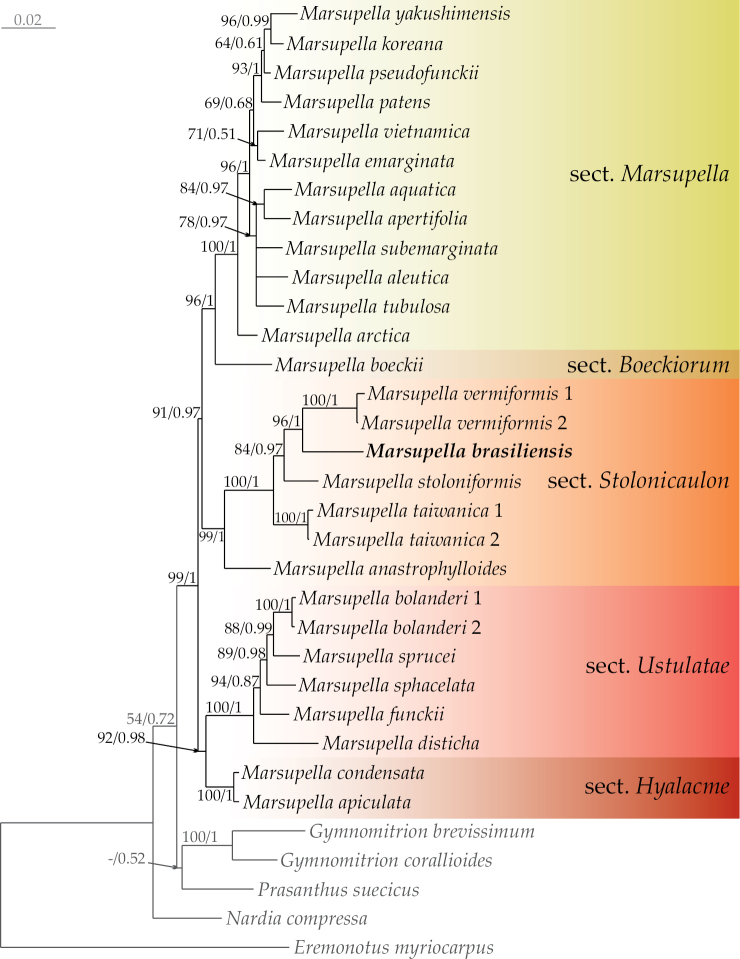
Phylogram obtained in a Bayesian analysis for the genus *Marsupella* based on the ITS 1‒2+*trn*L‒F dataset. The values of bootstrap support from the ML analysis and Bayesian posterior probabilities greater than 0.50 (50%) are indicated. The newly obtained sequences are marked in bold. Specimen voucher details and GenBank accession numbers are listed in Table 1.

**Figure 2.**
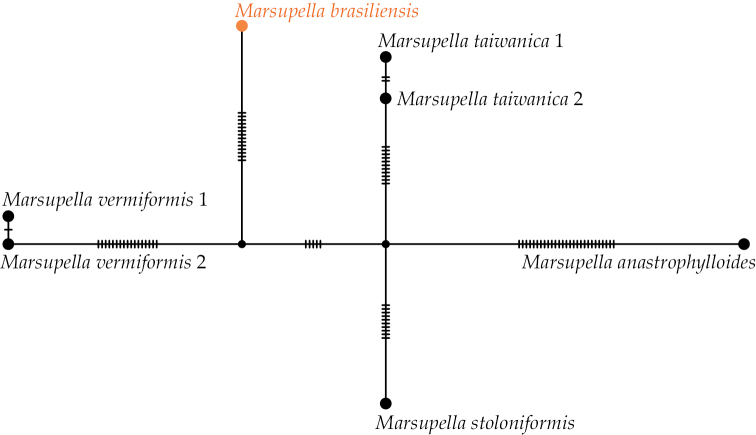
TCS haplotype network of ITS 1‒2+*trn*L‒F sequences for Marsupellasect.Stolonicaulon. Dashes indicate the number of nucleotide substitutions. Specimen voucher details and GenBank accession numbers are listed in Table 1.

On the Bayesian phylogenetic tree (Fig. 1), which demonstrates the ML topology with an indication of bootstrap support values (BS) and Bayesian posterior probabilities (PP), specimen ASV15033 formed a clade within sect. Stolonicaulon with high support of BS = 96% and PP = 1 (or 96/1). The TCS haplotype network (Fig. 2) revealed five haplotypes separated from each other by many nucleotide substitutions (at least 10), and specimen ASV15033 formed a separate group.

The morphological comparison confirmed many traits indicating that the studied specimen belongs to sect. Stolonicaulon. Along with the latter, the comparison showed some features that do not fit well with the morphology of *M.microphylla* (Schuster 1978, 1996); the most valuable features are stem cross-section, leaf and sporophyte characteristics. The latter, along with the results of molecular genetic comparison, led to the conclusion that the studied specimen belongs to Marsupellasect.Stolonicaulon; however, it is not conspecific with ‘true’ *M.microphylla*, as it was originally identified. Below, we provide the corresponding description of the specimen along with a discussion on its morphological traits.

### ﻿Taxonomy

#### ﻿ Marsupella brasiliensis

Taxon classificationPlantaeJungermannialesGymnomitriaceae

Bakalin, Maltseva & Schäf.-Verw. sp. nov.

0FE0A530-DCBE-57C1-AA3F-D5775D7E7E5F

##### Description.

Plants wiry, ascending in loose patches, densely intermixed with *Cephaloziella* sp. and *Metasolenostoma* sp., perianthous plants strongly clavate, from densely branched and rhizomatous base, sterile branches 120–200 µm wide with leaves, in perichaetium zone to 900 µm wide, rusty to brownish and grading to whitish brown in older parts, with red tint in the leaf apices in the apical part of shoot. Rhizoids virtually absent in leaved shoots, in rhizomatous base sparse and colorless, separated (not united to the fascicles). Stem 70–90 µm in diameter, branching ventral, but new branch always arose near to the ventral base of the leaf and may be regarded as deeply postical-intercalary; cross section nearly orbicular, outer cells with outer wall thin, other slightly thickened (10-)12–14 µm in diameter, inward cell walls become thicker, with large triangular trigones, while cell size become smaller, 8–10 µm in diameter. Leaves obliquely to suberect spreading, slightly narrower to twice wider than stem, 50–180×60–190 µm, reniform to widely ovate, smaller constantly entire, sometimes with obtuse apex, larger divided by U-shaped sinus descending to 1/10–1/5 of leaf length, lobes acute. Underleaves absent. Midleaf cells strongly collenchymatous with large and slightly convex trigones, 10–14 µm in diameter to shortly oblong 10–16×8–12 µm, cuticle virtually smooth. Paroicous. Perianthous branches distinctly clavate, perigynium high, ca. 1250×550 µm, with 3 pairs of leaves whose lower pair composed by strongly ventricose bracts containing antheridia; perianth hidden within bracts, conical, eroding from the mouth, and completely disappearing after sporophyte emergence. Female bracts slightly wider than long, to 500×550 µm with gamma-shaped sinus descending to 1/4–1/3 of bract length, lobes triangular, acute, with somewhat diverging apices; bracteoles absent. Androecial bracts in 1(-2) pairs in lower part of perigynium, monandrous. Capsule wall bistratose, outer cells with nodular thickenings present on vertical walls and only sometimes present in horizontal walls; inner layer of rectangular cells with small nodular (not semicircular) thickenings. Spores brownish, 8–10 µm in diameter, faintly papillose, elaters bispiral, (-75)100–130×7–8 µm brown, with narrow, but never homogenous ends.

##### Type.

Brazil, Rio de Janeiro, ca. 22°24'S, 44°41'W, Serra de Itatiaia, Hochgebirgsvegetation auf der Hochfläche bei Abrigo Rebouças, an exponierter, zeitweise sickerfeuchter Felswand [Serra de Itatiaia, high alpine vegetation on the plateau near Abrigo Rebouças, on exposed, intermittently dripping cliff], 2420 m, 15. Oct. 1991, leg. Schäfer-Verwimp & Verwimp 15033 (holotype JE!; isotype VBGI!, PRC, SP (not restudied in the preparation of the present account)).

##### Illustration in present paper.

Fig. 3.

**Figure 3.**
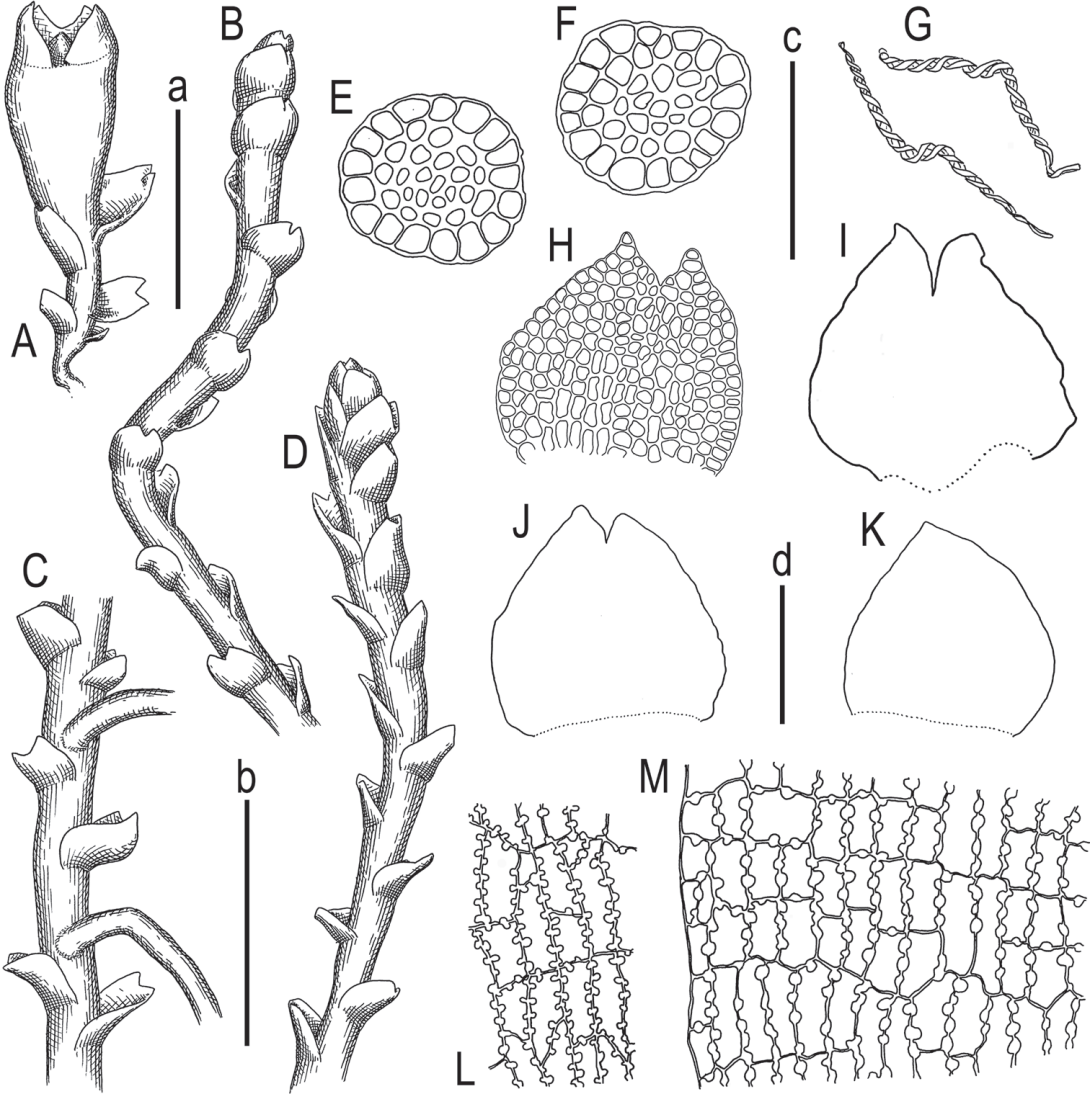
*Marsupellabrasiliensis* Bakalin, Maltseva et Schäf.-Verw. sp. nov. **A** perianth bearing shoot, fragment **B** plant habit, fragment, lateral view **C** plant habit, fragment, ventral view **D** plant habit, fragment, dorsal view **E, F** stem cross-section **G** elaters **H–K** leaves **L** capsule, inner layer **M** capsule, outer layer. Scale bars: 1 mm (a: **A**); 500 µm (b: **B–D, I**); 100 µm (c: **E–G, L, M**); 100 µm (d: **H–K**). All from 15033 (isotype VBGI).

## ﻿Discussion

The newly described *Marsupellabrasiliensis* seems to be a rather rare species, hitherto known only from the Itatiaia mountains of Southeast Brazil between 2300–2420 m. Váňa (1999) reported this specimen for the first time in Brazil under the name *M.microphylla* (type of *M.brasiliensis*), and subsequently, a second specimen was cited from the same region by Gradstein and Costa (2003), who found it growing together with *Cephalozielladivaricata* (Sm.) Schiffn. In the type specimen, shoot fragments of a *Metasolenostoma* Bakalin & Vilnet species have been detected (confirmed genetically as the new species of the latter genus, unpublished). *Marsupellabrasiliensis* was found only along the main road in the National Park on exposed rock faces, and further associated species are not known to us. It may have been overlooked due to its very small size and may rarely occur elsewhere around Itatiaia or Serra da Mantiqueira. Climate change may pose a threat to high-altitude bryophytes confined to Southeast Brazil.

The present study showed that this specimen identified as *Marsupellamicrophylla* previously and treated here as *M.brasiliensis* belongs to Marsupellasect.Stolonicaulon; therefore, the distribution of the section in Latin America is definitely confirmed. At the same time, the morphological study of the material revealed that the plants are not conspecific with *M.microphylla*, which has been described from the Venezuelan Andes (Estado Merida, Sierra Nevada de Merida ca 4160 m a.s.l.). On one side, the high peaks of Itatiaia and the “páramo” characteristic of the “Campos de Altitude” benefit the establishment of large numbers of Andean species, such as *Aureolejeuneafulva* R.M. Schust., *Diplasiolejeuneapauckertii* (Nees) Steph. (also present in Central America), *Drepanolejeuneagranatensis* (J.B. Jack & Steph.) Bischler, *Herbertusoblongifolius* (Steph.) Gradst. et Cleef, and others (Gradstein and Costa 2003; Costa et al. 2015, 2018). Macroclimatic congruities form the basis for strong Andean-southeast Brazilian biogeographic connections, which was also confirmed by Frahm (1991) in the moss genus *Atractylocarpus* Mitt. and *Campylopus* Brid. On another side, the Serra da Mantiqueira, including the Itatiaia region, is famous for its high number of endemic species, including *Coluraitatyana* Steph. and *Brachydontiumnotorogenes* W.R. Buck & Schäf.-Verw., as well as the genera *Cladastomum* Müll. Hal., *Crumuscus* W.R. Buck & Snider, and *Itatiella* Smith (Costa et al. 2018). Therefore, the nonspecificity of the Brazilian plant with the type of *Marsupellamicrophylla* is not truly surprising. Váňa (1999) mentioned differences in some sporophyte characters; however, he failed to describe a new species based on different seta structures alone. During investigation of the type specimen, some additional morphological differences came to light. Schuster (1996: 76) notes, “Elaters [in *M.microphylla*] very short rather stiff and sinuous, 6–7×60–70 µm, with 3 narrow spirals”, in addition, as seen in fig. 14:11 in l.c., the elater endings are abruptly rounded. Whereas *M.brasiliensis* described here possesses distinctly bispiral elaters, they are narrowed at the end. Second, although the general tendency of the stem structure characteristic for several taxa of sect. Stolonicaulon (as well as many *Marsupella* Dumort. species in general) is retained: larger and thin-walled (or thinner-walled) cells on the outer layer and smaller and thick-walled cells inward, the cell size of *M.brasiliensis* is much smaller. In the latter, the outer cells are 12–14 µm in diameter and the inner cells are 8–10 µm in diameter, versus 12–25 µm in the outer layer and 12–15 µm in the inner layer in *M.microphylla*. Third, the leaves of *M.microphylla* are consistently bilobed, while in our species, they are entire on smaller shoots and are only shortly incised on larger shoots. In addition, Váňa (1999) noted the difference in the seta cross section between the *M.microphylla* type and the specimens described here as *M.brasiliensis*. He found that the seta cross section consists of 10–14(-16) rows of cells in the outer layer with 4–8 rows in the inner layer, whereas that of *M.microphylla* possesses 8–10 outer rows and 3–4 inner rows. Váňa (1999) treated this variability in seta cross section as hardly valuable for the taxonomy of *Marsupella*.

Within Marsupellasect.Stolonicaulon, based on taxa whose position within the section is genetically confirmed, sporophytes are only known in *M.stoloniformis* N. Kitag. The elaters of this species are 2–3-spiral (Bakalin et al. 2022). Elaters of *Marsupellabrasiliensis* are strictly 2-spiral. It is worth mentioning that Váňa (1999: 226) wrote about the specimen discussed in the present study: “some elaters are 2-spiral (Schuster (1996) described as 3-spiral in *M.microphylla*)”. Therefore, it is unclear if Váňa actually saw the 3-spiral elaters or was referring to the 3-spiral elaters previously described by Schuster (1996). The capsule structure is very similar to that described for *M.microphylla*: capsule wall bistratose, with nodular thickening in both the outer and inner layers. Within sect. Stolonicaulon, *M.brasiliensis* is distinguished from other species by paroicous inflorescence (shares this feature with conditionally referred to sect. *StolonicaulonM.microphylla*, from which it immediately differs in bispiral elaters presence). The morphology of sterile plants of *M.brasiliensis* is similar to that of *M.vermiformis* (R.M. Schust.) Bakalin & Fedosov and *M.microphylla*; the haplotype network (Fig. 2) also shows the genetic relationship with *M.vermiformis*. *Marsupellabrasiliensis* differs from both species in its less deep and inconsistently incised leaves. The cell size of the leaves and the leaf cell network are similar in *M.brasiliensis* to those observed in *M.vermiformis* but not similar to those in *M.microphylla* (the cells are much smaller, as noted above). The stem cross-section characteristics of *M.vermiformis* are distinctly different from those of *M.brasiliensis* (smaller toward the margin versus distinctly larger toward the margin (cf. (Bakalin et al. 2019)), but somewhat similar to the stem cross-section characteristics of *M.microphylla*, *M.praetermissa* Bakalin et Vilnet and *M.anastrophylloides* Bakalin, Vilnet & Maltseva (cf. (Bakalin et al. 2022)).

Therefore, the present study has confirmed the occurrence of Marsupellasect.Stolonicaulon in Latin America, but was unable to determine whether *M.microphylla* belongs to sect. Stolonicaulon. Taking into account that the number of elater spirals and the shape of the elaters may be a rather important taxonomic feature, there are doubts that *M.microphylla* can be referred to as the same section, and the subgenus Nanocaulon may require re-evaluation, at least at the rank of section. Marsupellasubg.Nanocaulon was synonymized with M.sect.Boeckiorum Müll. Frib. ex R.M. Schust. (M.sect.Boeckiae Müll. Frib.) in Váňa (1999: 226), based on the fact that the differences between two sections are not sufficient to maintain them as separate entities: “At the moment I do not agree with the separation of infrageneric taxa only on the basis of a different seta structure”. This question also remained unanswered in the present research because we did not study molecularly ‘true’ *M.microphylla*. However, taking into account data in an integrative systematic study by Bakalin et al. (2019, 2022), where sect. Boeckiorum is strictly different from sect. Stolonicaulon, and considering the distribution pattern, we suggest that even if *M.microphylla* does not belong to sect. Stolonicaulon it also seems hardly possible that *M.microphylla* belongs to sect. Boeckiorum, which contains north Holarctic taxa and has a sister position to the Marsupellasect.Marsupella, which is also composed of North Holarctic species.

*Marsupellabrasiliensis* is thus far the only known species of the sect. Stolonicaulon with paroicous inflorescence and occurring in Latin America whose position has been confirmed genetically. All other species are dioicous (with adjustment for the fact that the inflorescences are not known for *M.praetermissa* and *M.anastrophylloides*). Finally, we may assume that the mountain systems of Latin America may hide additional taxa belonging to Marsupellasect.Stolonicaulon, and purposeful research in this field should be continued.

## Supplementary Material

XML Treatment for Marsupella brasiliensis
